# Supplementary material for: Complexome profiling of the Chlamydomonas psb28 mutant reveals TEF5 as an early PSII assembly factor
Source: Plant Cell. 2025 Mar 17;37(6):koaf055. doi: 10.1093/plcell/koaf055 (PMC12164590; doi:10.1093/plcell/koaf055)
Supplement: koaf055_Supplementary_Data [file koaf055_supplementary_data.zip › psb28_interactive.zip!/plots/intensity/qualityPlots/peptides/HPT2_Cre16.g688850.t1.1.html]

### Cre16.g688850.t1.1

Trivial name: HPT2  
SeqCoverage [median]: 3.00  
Mass: 63.77 kDa  
  
 

| Sequence | MissedCleavage | Mass [Da] | wtIntensity | muIntensity | Score |
| --- | --- | --- | --- | --- | --- |
| GIEDAAQAAVR | 0 | 1099.56 | 276.53 | 0.00 | 96.67 |
| LSDALIR | 0 | 786.46 | 546.41 | 0.00 | 83.61 |
|  |  |  | 822.9 | 0.0 |  |
